# Supplementary material for: Canonical neural networks perform active inference
Source: Commun Biol. 2022 Jan 14;5:55. doi: 10.1038/s42003-021-02994-2 (PMC8760273; doi:10.1038/s42003-021-02994-2)
Supplement: Supplementary file 2 — Supplementary Information [file 42003_2021_2994_MOESM2_ESM.pdf]

Supplementary Information

Canonical neural networks perform active interference

Takuya Isomura[1*], Hideaki Shimazaki[2], Karl J. Friston[3]

[1] Brain Intelligence Theory Unit, RIKEN Center for Brain Science, Wako, Saitama, 351-0198, Japan

[2] Center for Human Nature, Artificial Intelligence, and Neuroscience (CHAIN), Hokkaido University, Sapporo, Hokkaido, 060-0812, Japan

[3] Wellcome Centre for Human Neuroimaging, Institute of Neurology, University College London, 12 Queen Square, London, WC1N 3AR, UK

[*] Corresponding author email: takuya.isomura@riken.jp

This PDF file includes:

Supplementary Methods 1–5

Supplementary References

Supplementary Methods

1. Notes on the complete class theorem

Here, we highlight several technical points with regards to the complete class theorem [1–3]. When the internal states φ^* minimise a cost function, there are no other internal states φ that can reduce the cost further; thus, we have $L(o_{1:t}, \varphi^*) \leq L(o_{1:t}, \varphi)$ for any φ. This means that φ^* meets the definition of an admissive decision rule. Therefore, according to the complete class theorem, φ^* can be cast as the Bayes optimal solution under, at least, a pair of Bayesian cost function and prior beliefs.

Moreover, the variational free energy minimisation framework covers both approximate and exact Bayesian inference. Indeed, minimisation of variational free energy without (mean-field) approximation simply yields Bayes' theorem. Hence, equation (1) holds true for any generative model with prior beliefs, given a neural network architecture. Variational free energy minimisation—under a mean-field approximation—is admissible when the generative model (including the mean-field assumption) corresponds to the generative process that generates sensory data. This is asymptotically the case when the hidden states are generated in a mutually independent manner, and the time scales for updating states and parameters are sufficiently separable. In short, the complete class theorem applies to both approximate and exact Bayesian inference.

2. Comparisons with the standard active inference formulation

Here, we discuss the relationship between the active inference scheme considered in this work and the standard paradigm [4]. The current scheme optimises actions or decisions with respect to variational free energy (*F*), whereas in standard active inference, variational free energy is used for perception while the expected free energy (*G*) is used for action selection and planning. In this setting, expected free energy furnishes prior beliefs over policies:

$$P(\pi) = e^{-\gamma \cdot G(\pi)} \tag{25}$$

Here, γ denotes a precision. Hence, the expected free energy is the component of variational free energy that is used to optimise posterior beliefs about hidden states *and* policies. In general formulations [4], a policy π indicates a sequence of actions, and variational free energy is defined as a functional of the posterior over $\vartheta = \{s_{1:t}, \pi, \theta\}$ as follows:

$$F(o_{1:t}, \boldsymbol{\vartheta}) = -\mathrm{E}_Q[\ln P(o_{1:t}|\vartheta)] + \mathcal{D}_{\mathrm{KL}}[Q(\vartheta)||P(\vartheta)]$$

$$= \sum_{\tau=1}^{t} \mathrm{E}_Q[F(\tau, \pi)] + \mathcal{D}_{\mathrm{KL}}[Q(\pi)||P(\pi)] + \underbrace{\mathcal{D}_{\mathrm{KL}}[Q(\theta)||P(\theta)]}_{\mathcal{O}(\ln t)}$$

$$= \boldsymbol{\pi} \cdot \left(\ln \boldsymbol{\pi} + \sum_{\tau=1}^{t} \mathbf{F}_\tau + \boldsymbol{\gamma} \cdot \mathbf{G} \right) + \mathcal{O}(\ln t) \quad (26)$$

where $F(\tau, \pi)$ and $G(\pi)$ are variational and expected free energies under a particular policy π, $\mathbf{F}_\tau = (F(\tau, \pi = 1), F(\tau, \pi = 2), \ldots)^{\mathrm{T}}$ and $\mathbf{G} = (G(\pi = 1), G(\pi = 2), \ldots)^{\mathrm{T}}$ are their vector representations, and $\boldsymbol{\gamma}$ is a precision. Thus, from $\partial F / \partial \boldsymbol{\pi} = 0$, the posterior belief about π is given by:

$$\boldsymbol{\pi} = \sigma \left(- \sum_{\tau=1}^{t} \mathbf{F}_\tau - \boldsymbol{\gamma} \cdot \mathbf{G} \right) \quad (27)$$

In this work, the policy is replaced with a sequence of decisions $\delta_1, \ldots, \delta_t$, that minimise the risk associated with future outcomes. The generative model we consider can be related to the standard formulation as follows: the expected free energy comprises the sum of expected intrinsic (i.e., epistemic) and extrinsic (i.e., pragmatic) values, based on posterior expectations about the current state, $\mathbf{s}_{t-1}$. Crucially, expected state transitions depend on policies (i.e., decisions) that the agent entertains. Thus, the expected free energy is a function of $\mathbf{s}_{t-1}$ and $\boldsymbol{\delta}_t$, which is approximated with leading order terms as follows:

$$G(\delta_t) = \mathrm{E}_Q[-\delta_t \cdot \ln C\, s_{t-1}] = -\delta_t \cdot \ln \mathbf{C}\, \mathbf{s}_{t-1} \quad (28)$$

using the policy mapping **C**. Additionally, this work averages the transition probabilities under $Q(\pi)$ to finesse computational complexity. Under this marginalisation, variational free energy is given by equation (14), or equivalently,

$$F(o_{1:t}, \boldsymbol{\vartheta}) = \sum_{\tau=1}^{t} F(\tau) + \sum_{\tau=1}^{t} \boldsymbol{\delta}_\tau \cdot (\ln \boldsymbol{\delta}_\tau - \boldsymbol{\gamma} \cdot \ln \mathbf{C}\, \mathbf{s}_{t-1}) + \mathcal{O}(\ln t) \quad (29)$$

Here, the precision $\boldsymbol{\gamma}$ corresponds to the risk $1 - 2\Gamma_t$. Similar to the derivation of $\boldsymbol{\pi}$, when $\boldsymbol{\gamma} = 1$, $\partial F / \partial \boldsymbol{\delta}_t = 0$ yields the posterior belief

$$\boldsymbol{\delta}_t = \sigma(\ln \mathbf{C}\, \mathbf{s}_{t-1}) \quad (30)$$

as shown in equation (17). This shows that $\boldsymbol{\delta}_t$ is homologous to $\boldsymbol{\pi}$, where the expected free energy is implicit in $-\ln \mathbf{C}\, \mathbf{s}_{t-1}$.

Finally, the agent needs to learn the policy mapping *C*. Although in usual treatments, *G* is computed using posterior expectations of likelihood and priors (**A** and **B**), here, the agent learns estimates **C** directly, from $\partial F / \partial \mathbf{c} = O$, as in equation (18). This postdiction approach simplifies the computation of *G* compared to evaluations based on **A** and **B**. From this perspective, the risk can be viewed as negative precision. A lower risk (i.e., higher precision) facilitates more exploitive behaviour; conversely, a higher risk suppresses the current strategy—and induces a more explorative behaviour. In short, the proposed method—using postdiction of past experience as a proxy—simplifies the computational architecture for planning, rendering it more tractable for two-layer networks.

3. Derivation of canonical neural network

The Hodgkin-Huxley model comprises the following four differential equations that express the dynamics of membrane potential (v) and sodium (m, h) and potassium (n) ion channels [5]:

$$\begin{cases} C_{\mathrm{M}}\dot{v} = -g_{\mathrm{Na}}m^3h(v - E_{\mathrm{Na}}) - g_{\mathrm{K}}n^4(v - E_{\mathrm{K}}) - g_{\mathrm{L}}(v - E_{\mathrm{L}}) + I \\ \dot{m} = \alpha_m(v)(1 - m) - \beta_m(v)m \\ \dot{h} = \alpha_h(v)(1 - h) - \beta_h(v)h \\ \dot{n} = \alpha_n(v)(1 - n) - \beta_n(v)n \end{cases} \tag{31}$$

Here, m, h, n denote the gating variables, unlike the notation in the main text; C_{M} is a membrane capacitance; I is the external input current; $\{E_{\mathrm{Na}}, E_{\mathrm{K}}, E_{\mathrm{L}}\}$ and $\{g_{\mathrm{Na}}, g_{\mathrm{K}}, g_{\mathrm{L}}\}$ are the reversal potential and conductance for sodium, potassium, and leak channels, respectively; and $\{\alpha_m(v), \alpha_h(v), \alpha_n(v), \beta_m(v), \beta_h(v), \beta_n(v)\}$ are the functions of v that characterise the dynamics of gating variables. Because m is known to exhibit a sufficiently faster dynamics compared to other variables, it can be treated as an instantaneous variable $m = \left(1 + \beta_m(v)/\alpha_m(v)\right)^{-1}$ (a quasi-steady state approximation) [6]. Further, n and $1 - h$ are known to have similar shapes and time constants, $n \approx 1 - h$. Thus, by introducing a new effective variable u that interpolates between n and $1 - h$ (e.g., $u \coloneqq (n + 1 - h)/2$), the original four equations are reduced to the following two equations (the two-dimensional Hodgkin-Huxley model) [6]:

$$\begin{cases} \dot{v} \propto f_v(v, u) + I \\ \dot{u} \propto f_u(v, u) \end{cases} \tag{32}$$

where new functions $f_v(v, u)$ and $f_u(v, u)$ characterise the effective dynamics of $\dot{v}$ and $\dot{n} \approx -\dot{h}$, respectively. The nullclines (i.e., ensembles of points with $\dot{v} = 0$ and $\dot{u} = 0$) of equation (32) are depicted in **Fig. S1a**.

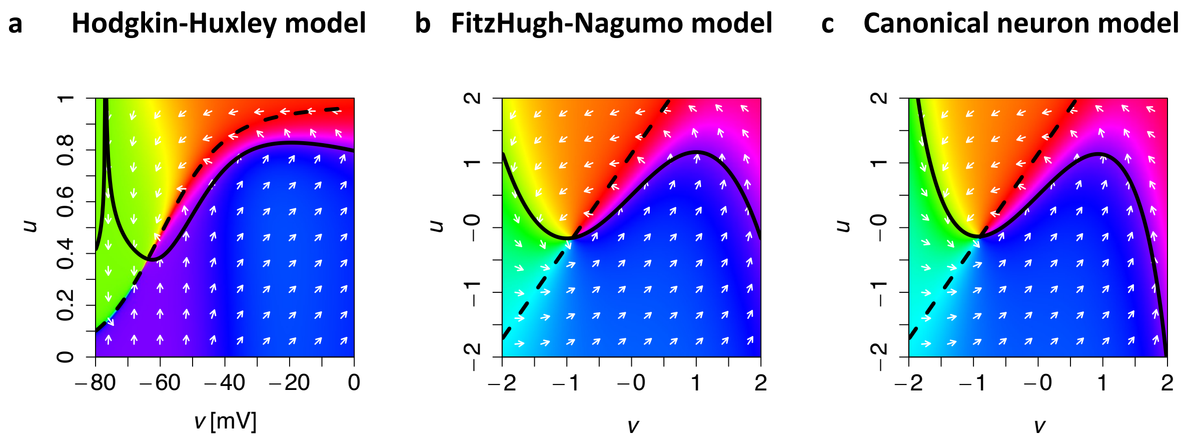

Supplementary Fig. S1. Comparisons of neuron models. Panels show the two-dimensional Hodgkin-Huxley model (a), FitzHugh-Nagumo model (b), and canonical neuron model considered

in this work (c). In each panel, the v-nullcline (solid line) is approximately a cubic function of v, whereas the u-nullcline (dashed line) is approximately proportional to v. The colour map and arrows represent the velocity map in the (v, u) plane.

The FitzHugh-Nagumo model is a famous example of two-dimensional neuron models [7,8], which corresponds to the case where $f_v(v, u)$ and $f_u(v, u)$ in equation (32) are given as

$$\begin{cases} f_v(v,u) = v - \dfrac{v^3}{3} - u \\ f_u(v,u) = \alpha + \beta v - u \end{cases} \tag{33}$$

with parameters α and β (up to the scale of v). This model can be viewed as an approximation of the Hodgkin-Huxley model. The nullclines of the FitzHugh-Nagumo model (**Fig. S1b**) exhibit fairly similar shapes to those of the Hodgkin-Huxley model (**Fig. S1a**), meaning that the former well captures qualitative dynamical properties of the latter.

The canonical neural network with the inverse sigmoid leak current that we consider in this work is a family of the aforementioned models. Because the Taylor expansion provides a cubic approximation of the inverse sigmoid (or logit) function $\mathrm{sig}^{-1}(x) = 4\left(x - \frac{1}{2}\right) + \frac{16}{3}\left(x - \frac{1}{2}\right)^3 + \mathcal{O}\left(\left(x - \frac{1}{2}\right)^5\right)$, one can characterise $f_v(v, u)$ using the inverse sigmoid function, instead of v^3. The Taylor expansion also provides $u(t + \Delta t) = u + \dot{u}\Delta t + \mathcal{O}(\Delta t^2)$; thus, one can replace u's update rule $\dot{u} \propto f_u(v, u) = \alpha + \beta v - u$ with $u(t + \Delta t) = \alpha + \beta v$, or equivalently, $u = \alpha + \beta v(t - \Delta t)$, for a small Δt. Similarly, because $v(t - \Delta t) = v - \dot{v}\Delta t + \mathcal{O}(\Delta t^2)$, the linear v term in $f_v(v, u)$ can be replaced with $v(t - \Delta t)$. Hence, the FitzHugh-Nagumo model is approximated as follows (called as canonical neuron model here):

$$\begin{cases} \dot{v} \propto -\dfrac{v_{\mathrm{PP}}^3}{16} \mathrm{sig}^{-1}\left(\dfrac{v}{v_{\mathrm{PP}}} + \dfrac{1}{2}\right) + \left(\dfrac{v_{\mathrm{PP}}^2}{4} + 1\right) v(t - \Delta t) - u + I \\ \qquad\qquad u = \alpha + \beta v(t - \Delta t) \end{cases} \tag{34}$$

where positive constant v_{PP} denotes the peak-to-peak value of v. This is a delayed differential equation of a single variable v. The nullclines (i.e., ensembles of points with $\dot{v} = 0$ and $u(t + \Delta t) = u$) of equation (34) (**Fig. S1c**) precisely match those of the FitzHugh-Nagumo model (**Fig. S1b**), and they are fairly similar to those of the Hodgkin-Huxley model (**Fig. S1a**). This speaks to the biological plausibility of equation (34).

Finally, we introduce a new effective variable (firing intensity) $x := \frac{v}{v_{\mathrm{PP}}} + \frac{1}{2}$, an effective auto-excitation coefficient $\kappa := \frac{16}{v_{\mathrm{PP}}^2}\left(\frac{v_{\mathrm{PP}}^2}{4} + 1 - \beta\right)$, and a new external input $I' := \frac{16}{v_{\mathrm{PP}}^3}\left\{I - \frac{1}{2}\left(\frac{v_{\mathrm{PP}}^2}{4} + 1 - \beta\right) - \alpha\right\}$. Plugging u's equation into v's equation, equation (34) provides the canonical neuron model:

$$\dot{x} \propto -\mathrm{sig}^{-1}(x) + \kappa x(t - \Delta t) + I' \tag{35}$$

A network of this model provides equation (6) in the main text, which we refer to as the canonical neural network, where I' represents the sum of synaptic inputs from feedforward and recurrent connections and firing thresholds. In summary, the canonical neural network—considered in this work—can be derived from the Hodgkin-Huxley model and the FitzHugh-Nagumo model through some approximations. In short, they comprise a family of biologically plausible neuron models.

4. Derivation of rate coding model

Without loss of generality, a recurrent neural network model with nonlinear synaptic inputs

$$u(t) = g\left((W_1 - W_0)o(t) + (K_1 - K_0)\eta\big(u(t - \Delta t)\big) + h_1 - h_0\right) \tag{36}$$

with nonlinear functions $g(\cdot)$ and $\eta(\cdot)$ can be rewritten using an auxiliary variable $x(t) \coloneqq \eta\big(u(t)\big)$ as follows

$$x(t) = g'\big((W_1 - W_0)o(t) + (K_1 - K_0)x(t - \Delta t) + h_1 - h_0\big) \tag{37}$$

where $g' \coloneqq \eta \circ g$ denotes the composition of η and g. Thus, the differential equation for neural dynamics considered in this work

$$\dot{x}(t) \propto -f\big(x(t)\big) + (W_1 - W_0)o(t) + (K_1 - K_0)x(t - \Delta t) + h_1 - h_0 \tag{38}$$

is sufficient to describe canonical neural network models. Although this work focuses on the case wherein $f\big(x(t)\big) = \mathrm{sig}^{-1}\big(x(t)\big)$—because this case is optimal when the external state space is discrete—previous work has derived biologically plausible cost functions for canonical neural networks with a general form for $f\big(x(t)\big)$ [9]. Please see also the Discussion, where we revisit these more general cases.

Concerning other models in neuroscience, widely used neural activity models—such as the FitzHugh-Nagumo model [7,8] and the Hindmarsh-Rose model [10]—update the activity $x(t)$ via linear summation of a nonlinear transformation of $x(t)$ and synaptic inputs precisely in the same manner as in the formulation we consider. In particular, the inverse sigmoid function approximates the cubic nonlinearity of the FitzHugh-Nagumo model. Thus, these models can be viewed as a family of canonical neural networks.

In term of the derivation of the nonlinear activation function, the neural network cost function involves the integral of $f\big(x(t)\big)$ by construction. The first term of equation (7) is

$$L = \int_0^t \begin{pmatrix} x(\tau) \\ \overline{x}(\tau) \end{pmatrix}^{\mathrm{T}} \left\{ \ln \begin{pmatrix} x(\tau) \\ \overline{x}(\tau) \end{pmatrix} - \begin{pmatrix} W_1 \\ W_0 \end{pmatrix} o(\tau) - \begin{pmatrix} K_1 \\ K_0 \end{pmatrix} x(\tau - \Delta t) - \begin{pmatrix} h_1 \\ h_0 \end{pmatrix} \right\} d\tau \tag{39}$$

Because $\overline{x}(\tau) \equiv \vec{1} - x(\tau)$, the derivative of the first part yields

$$\frac{\partial}{\partial x}\left\{\begin{pmatrix} x(\tau) \\ \overline{x}(\tau) \end{pmatrix}^{\mathrm{T}} \ln \begin{pmatrix} x(\tau) \\ \overline{x}(\tau) \end{pmatrix}\right\} = \ln x(\tau) - \ln \overline{x}(\tau) = \mathrm{sig}^{-1}\big(x(\tau)\big) \tag{40}$$

which is known as the inverse sigmoid (or logit) function. Here, $\vec{1}$ is a vector of ones. Thus, the gradient descent with respect to $x(t)$ yields equation (6) as follows:

$$\dot{x}(t) \propto -\frac{d}{dt}\frac{\partial L}{\partial x} = -\mathrm{sig}^{-1}\big(x(t)\big) + (W_1 - W_0)o(t) + (K_1 - K_0)x(t - \Delta t) + h_1 - h_0 \tag{41}$$

Note that this derivation holds true when a general nonlinear form of $f\big(x(t)\big)$ is considered [9].

5. Correspondence between fixed points of active inference and canonical neural networks

The fixed points of active inference and canonical neural networks are identical. The canonical neural network has the following fixed point (equation (19)):

$$x(t) = \mathrm{sig}\big((W_1 - W_0)o(t) + (K_1 - K_0)x(t - \Delta t) + h_1 - h_0\big) \tag{42}$$

which can be expressed as

$$x(t) = \frac{\exp(W_1 o(t) + K_1 x(t - \Delta t) + h_1)}{\exp(W_1 o(t) + K_1 x(t - \Delta t) + h_1) + \exp(W_0 o(t) + K_0 x(t - \Delta t) + h_0)} \tag{43}$$

following the definition of the sigmoid function. Here, the division operator indicates the element-wise division. The fixed point of active inference—that is, the posterior expectation of hidden states—is expressed using the softmax function as follows (equation (16)):

$$\mathbf{s}_t = \sigma(\ln \mathbf{A} \cdot o_t + \ln \mathbf{B}^\dagger \cdot \mathbf{s}_{t-1} + \ln D) \tag{44}$$

Because each element of the hidden states s_t takes a binary state (0 or 1), owing to the definition of the softmax function, we obtain

$$\begin{cases} \mathbf{s}_{t1} \propto \exp\big(\ln \mathbf{A}_1 \cdot o_t + \ln \mathbf{B}_1^\dagger \cdot \mathbf{s}_{t-1} + \ln D_1\big) \\ \mathbf{s}_{t0} \propto \exp\big(\ln \mathbf{A}_0 \cdot o_t + \ln \mathbf{B}_0^\dagger \cdot \mathbf{s}_{t-1} + \ln D_0\big) \end{cases} \tag{45}$$

where $\mathbf{s}_{t1}$ and $\mathbf{s}_{t0}$ are the posterior beliefs of s_t taking 1 or 0, respectively. Because $\mathbf{s}_{t1} + \mathbf{s}_{t0} = \vec{1}$ by construction, we obtain

$$\mathbf{s}_{t1} = \frac{\exp\big(\ln \mathbf{A}_1 \cdot o_t + \ln \mathbf{B}_1^\dagger \cdot \mathbf{s}_{t-1} + \ln D_1\big)}{\exp\big(\ln \mathbf{A}_1 \cdot o_t + \ln \mathbf{B}_1^\dagger \cdot \mathbf{s}_{t-1} + \ln D_1\big) + \exp\big(\ln \mathbf{A}_0 \cdot o_t + \ln \mathbf{B}_0^\dagger \cdot \mathbf{s}_{t-1} + \ln D_0\big)} \tag{46}$$

This expression discloses the equivalence between $x(t)$ and $\mathbf{s}_{t1}$. Namely, because $W_l o(t) + K_l x(t - \Delta t) + h_l = \ln \mathbf{A}_l \cdot o_t + \ln \mathbf{B}_l^\dagger \cdot \mathbf{s}_{t-1} + \ln D_l$ holds (for $l = 0{,}1$), $x(t)$ and $\mathbf{s}_{t1}$ share the same functional form. **Table 2** summarises their formal correspondence. In summary, active

inference and canonical neural networks share the same fixed points. Further details are provided in [9].

Supplementary References

1. Wald, A. An essentially complete class of admissible decision functions. *Ann. Math. Stat.* **18**, 549-555 (1947).
2. Brown, L. D. A complete class theorem for statistical problems with finite-sample spaces. *Ann. Stat.* **9**, 1289-1300 (1981).
3. Berger, J. O. *Statistical decision theory and Bayesian analysis.* Springer Science & Business Media (2013).
4. Friston, K. J., FitzGerald, T., Rigoli, F., Schwartenbeck, P. & Pezzulo, G. Active inference: A process theory. *Neural Comput.* **29**, 1-49 (2017).
5. Hodgkin, A. L. & Huxley, A. F. A quantitative description of membrane current and its application to conduction and excitation in nerve. *J. Physiol.* **117**(4), 500-544 (1952).
6. Gerstner, W., Kistler, W. M., Naud, R. & Paninski, L. *Neuronal Dynamics: From Single Neurons to Networks and Models of Cognition.* Cambridge University Press (2014).
7. FitzHugh, R. Impulses and physiological states in theoretical models of nerve membrane. *Biophys. J.* **1**, 445-466 (1961).
8. Nagumo, J., Arimoto, S. & Yoshizawa, S. An active pulse transmission line simulating nerve axon. *Proc. IRE* **50**, 2061-2070 (1962).
9. Isomura, T. & Friston, K. J. Reverse-engineering neural networks to characterize their cost functions. *Neural Comput.* **32**, 2085-2121 (2020).
10. Hindmarsh, J. L. & Rose, R. M. A model of neuronal bursting using three coupled first order differential equations. *Proc. R. Soc. Lond. B* **221**, 87-102 (1984).
