# Supplementary material for: Canonical neural networks perform active inference
Source: Commun Biol. 2022 Jan 14;5:55. doi: 10.1038/s42003-021-02994-2 (PMC8760273; doi:10.1038/s42003-021-02994-2)
Supplement: Supplementary file 3 — Description of Additional Supplementary Files [file 42003_2021_2994_MOESM3_ESM.pdf]

Description of Additional Supplementary Files

File name: Supplementary Data 1
Description: The source data to generate Figs. 4 and 5.
